# Developing neuropalliative care for sporadic Creutzfeldt-Jakob Disease

**DOI:** 10.1080/19336896.2022.2043077

**Published:** 2022-03-03

**Authors:** Krista L. Harrison, Sarah B. Garrett, Joni Gilissen, Michael J. Terranova, Alissa Bernstein Sideman, Christine S. Ritchie, Michael D. Geschwind

**Affiliations:** a Division of Geriatrics, University of California, San Francisco, USA; b Philip R. University of California, San Francisco, USA; c Global Brain Health Institute, University of California, San Francisco, California, USA; d End-of-Life Care Research Group, Department of Family Medicine & Chronic Care, Vrije Universiteit Brussel (Vub), Belgium; e Memory and Aging Center, Department of Neurology, Weill Institute for Neurosciences, University of California San Francisco, San Francisco, CA, USA; f Department of Humanities and Social Sciences, University of California San Francisco, San Francisco, California, USA; g The Mongan Institute and the Division of Palliative Care and Geriatric Medicine, Massachusetts General Hospital, Boston, USA

**Keywords:** Sporadic Creutzfeldt-Jakob, palliative, qualitative, mixed methods, caregiver

## Abstract

We aimed to identify targets for neuropalliative care interventions in sporadic Creutzfeldt-Jakob disease by examining characteristics of patients and sources of distress and support among former caregivers. We identified caregivers of decedents with sporadic Creutzfeldt-Jakob disease from the University of California San Francisco Rapidly Progressive Dementia research database. We purposively recruited 12 caregivers for in-depth interviews and extracted associated patient data. We analysed interviews using the constant comparison method and chart data using descriptive statistics. Patients had a median age of 70 (range: 60–86) years and disease duration of 14.5 months (range 4–41 months). Caregivers were interviewed a median of 22  (range 11–39) months after patient death and had a median age of 59 (range 45–73) years. Three major sources of distress included (1) the unique nature of sporadic Creutzfeldt-Jakob disease; (2) clinical care issues such as difficult diagnostic process, lack of expertise in sporadic Creutzfeldt-Jakob disease, gaps in clinical systems, and difficulties with end-of-life care; and (3) caregiving issues, including escalating responsibilities, intensifying stress, declining caregiver well-being, and care needs surpassing resources. Two sources of support were (1) clinical care, including guidance from providers about what to expect and supportive relationships; and (2) caregiving supports, including connection to persons with experience managing Creutzfeldt-Jakob disease, instrumental support, and social/emotional support. The challenges and supports described by caregivers align with neuropalliative approaches and can be used to develop interventions to address needs of persons with sporadic Creutzfeldt-Jakob disease and their caregivers.

## Introduction:

Prion diseases such as sporadic Creutzfeldt-Jakob disease (sCJD) are rare but devastating in their rapid progression to serious disability, profoundly impacting patients and their family caregivers [1]. sCJD is the most common form of human prion disease, with about 400 cases of sCJD in the USA annually [2]. Prion diseases develop when a cellular protein in the nervous system is misfolded and aggregates. Prion diseases occur through multiple mechanisms – spontaneously (e.g., sCJD); genetically, through autosomal dominant mutations in the prion protein gene (*PRNP*); and acquired/infectious, such as through exposure to contaminated surgical equipment used previously on a person who unknowingly had CJD. Persons with sCJD and their caregivers experience a high burden of suffering due to the patient’s rapid loss of cognition, coordination, control of motor function and general bodily-function [3]. In about 90% of persons with sCJD, death typically occurs within one year (4.4 to 14 months) of symptom onset [4,5], with a correct and clear diagnosis often coming about 2/3 of the way through the disease course [6]. This leaves persons with sCJD and caregivers little time to prepare for end-of-life care. Given the current lack of disease-altering treatments for sCJD, appropriate care focuses on symptom management and promoting quality of life for both persons with sCJD and their caregivers [7,8].

Neuropalliative care is an emerging subspeciality and an interdisciplinary approach to reducing suffering and improving quality of life for persons with neurological illnesses and caregivers [9,10]. Palliative care approaches include symptom management, emotional and spiritual support, and guidance about treatment decisions. There is international consensus around the importance of palliative care for persons with longer-course dementia syndromes [11]. Little is known, however, about utilizing palliative care to address needs in rapidly progressing dementias (RPDs), with limited literature on palliative care in prion disease [8,12–14]. In sCJD, management is difficult because the rapid decline results in the degree and type of symptoms occurring within a few months of onset that are comparable to advanced stages of other neurodegenerative dementias that progress over many years [7,8,15,16], Caregivers and families typically need help managing distress about treatment decisions, especially those with implications for life-extension (e.g. tube feeding) [8,12,14,17]. We are only aware of one study, by Ford et al. (2018), that has specifically focused on caregivers’ struggles to manage symptoms of persons with sCJD [7].

We aimed to expand on Ford et al.’s work by using a mixed methods study informed by a palliative care framework [18] to comprehensively explore a range of both challenges and sources of support among caregivers of persons who died from sCJD. To our knowledge, this is the first in-depth description of palliative care needs associated with sCJD. Our findings highlight opportunities to add palliative care approaches and tools into regular neurology care for prion disease, and opportunities to improve prion-specific care among palliative care and hospice clinicians. These insights may be applicable to other rare diseases or to longer-course neurodegenerative diseases as well.

## Results

Eight of 12 persons who died from sCJD had participated in a 2-day clinical research visit and 4 with limited contact with the RPD study team had been admitted to the UCSF inpatient neurology service (Table 1). Median age at first UCSF visit was 70 years old (range 60–86). All patients met UCSF, European 2009, and European 2017 diagnostic criteria for probable sCJD and included of a variety of molecular subtypes [15,19,20]. The median disease duration was 14.5 months (range 4–41); onset to UCSF visit was 8 months (range 1–25); and first UCSF visit to death was 2.5 months (range 0–26), indicating participants were a median of ¾ through their disease course at their visit. Of the 8 research patients, median assessments scores were consistent with moderate to severe dementia and moderate dependence for activities of daily living (ADLs); although less quantitative data was available on the four inpatients, they were typically more impaired (e.g. median time from diagnosis to death was 1 month [range 0–4) for those admitted to inpatient services versus 5 months (range 1–27) for research visit participants).

**Table 1.** Patient and caregiver characteristics

Sociodemographic	Patients^1^	Caregivers^2^
	N = 12 (%)	N = 12 (%)
Age at data collection (mean [SD]) (median [range])	71.4 (8.8) 70 (60–86)	59 (45–73)
Sex		
Female	7 (58)	6 (50)
Male	5 (42)	5 (42)
Race/Ethnicity^3^		
Asian	1 (8)	1 (8)
Latinx/Hispanic	1 (8)	0
White	10 (83)	9 (75)
Black/African American	0	0
Declined to report	0	1 (8)
Educational level		
Less than or equal to high school	5 (42)	0
High school to some college	3 (25)	2 (17)
College or graduate school	2 (17)	9 (75)
no record/declined	2 (17)	1 (8)
Marital status at time of data collection		
Married	9 (75)	6
Widowed	2 (17)	4
Divorced	0	1
Single	1 (8)	0
Income category (total household)		
$40,000 – <$60,000		2 (17)
$60,000 – <$80,000		1 (8)
$80,000 – <$100,000		1 (8)
$100,000+		6 (50)
Declined to report		2 (17)
Number of people in household (mean [SD]) (median [range])		2.5 (1.5) (1–6)
Patient Disease/health characteristics		
Data from full UCSF MAC RPD research study visit (n)	8 (70)	
Data primarily from inpatient records (n)	4 (30)	
Total disease duration from onset^4^ to death, months (mean [SD]) (median [range])	15.6 (11.7) 14.5 (4–41)	
Time between onset and diagnosis^5^, months (mean [SD]) (median [range])	9.1 (7.8) 6.5 (1–25)	
Time between onset and first UCSF visit, months (mean [SD]) (median [range])	10.1 (8.0) 8.0 (1–25)	
Time of UCSF visit as percentage of disease course (mean [SD]) (median [range])	68% (28%) 74% (25–100)	
Time between first UCSF visit to death, months (mean [SD]) (median [range]))	5.1 (7.4) 2.5 (0–26)	
Time from diagnosis to death, months (mean [SD]) (median [range]))	6.1 (7.9) 3.0 (0–27)	
Patient cognitive and physical functioning and symptoms at time of first visit at UCSF (n = 8 who participated in a 2-day outpatient research visit)		
Mini-Mental State Examination (MMSE)^6^, (mean [SD]) (median [range])	13.5 (8.9) 12.5 (0–25)	
Barthel Index^7^ (mean [SD]) (median [range])	66.3 (36.2) 77.5 (0–100)	
U.K. Medical Research Council (MRC) prion disease rating scale^8^ (mean [SD]) (median [range])	13 (5.4) 14 (3–19)	
Clinical Dementia Rating (CDR) scale^9^ sum of boxes score (mean [SD]) (median [range])	11.1 (5.2) 11 (3.5 − 18)	
Neuropsychiatric Inventory (NPI- Caregiver only)^10^ composite score (mean [SD]) (median [range])	9.3 (6.8) 6 (4–24)	
Geriatric Depression Scale (GDS) Long Form^11^ (mean [SD]) (median [range])	8.2 (5.6) 9 (2–15)	
Prion type & diagnostic tests^12^ (n = 12)		
*PRNP* analysis^13^ showed no mutation, *n*	12 (100)	
CODON129 type		
129 M/V	5 (42)	
129 V/V	3 (25)	
129 M/M	4 (33)	
MRI diagnostic for CJD	11^14^	
CSF RT-QuIC test positive for sCJD	11 (1 no LP)	
Brain autopsy positive for sCJD (definite sCJD)	11 positive ^15^	

Acronyms: sCJD, sporadic Creutzfeldt Jakob Disease; LP, lumbar puncture; MAC, Memory and Ageing Center; RPD, rapidly progressive dementia; UCSF, University of California, San Francisco

^1^Data on patients came from chart reviews of the UCSF MAC RPD database and UCSF MAC general research (‘LAVA’) databases, which include extensive information on persons with probable or definite sCJD who separately consented to the ongoing use of their data from medical records and/or from research records through an IRB-approved UCSF study of RPDs. For patients who participated in a 2-day outpatient research visit, data included assessments of cognition (mini-mental state examination [MMSE] [36], Clinical Dementia Rating [CDR] scale [37]); function (Medical Research Council [MRC] prion disease scale [39], Barthel Index [40], CDR scale), neuropsychiatric symptoms (Neuropsychiatric Inventory [NPI] [44]); mood (long form Geriatric Depression Scale [GDS-L]) [38]. For inpatients who were only seen in the UCSF clinical wards (and did not participate in the more extensive 2-day research visit), more limited data was extracted from their EPIC electronic health record and (if available) from the UCSF MAC databases. Sources of diagnostic information included brain tissue pathology, cerebrospinal fluid (CSF) biomarkers of neuronal cell injury (i.e.14-3-3 Western blot, total-tau and neuron specific protein levels [45]), CSF RT-QuIC analysis [41], our internal review of brain MRI(s), and prion protein gene (*PRNP*) analysis (done through the US National Prion Disease Pathology Surveillance Center, Cleveland, OH) [42]. Missing data: age at time of visit (n = 1); educational level (n = 1); MRC prion rating scale (n = 1); CDR scale (n = 1); NPI (n = 1); GDS scale (n = 3 because scale could not be administered due to patient factors); MRI (n = 1); RT-QuIC test (n = 1); brain autopsy (n = 1 because family refused).

^2^Demographic data on caregivers were self-reported in a survey completed after the interview [19,35]. We recruited caregivers from lists of main contacts for patients in the UCSF MAC RPD database who had died from sCJD at least 3 months but no more than 3 years previously. One caregiver refused the demographic survey.

^3^Race/ethnicity data collected to report per funder requirements. Caregiver data was self-reported and categorized based on NIH reporting categories.

^4^“Onset’ as identified by the treating neurologist in the patient file at the earliest symptom we could identify based on available medical records and/or the medical history obtained during our research visits

^5^Date of ‘diagnosis” at which sCJD became the leading and most likely as identified by the treating neurologist in the patient file (could have been at UCSF or elsewhere), based on own assessment, reports from other physicians send to neurologist or highlighted by caregiver or patient during UCSF visit.

^6^Mini-Mental State Examination (MMSE) total score range: 0–30, with higher scores indicting more normal cognition

^7^Barthel Index total score range: 0–100, with lower scores suggesting greater dependency on activities of daily living

^8^Medical Research Council (MRC) prion disease rating scale^4^, total 0–20, with lower scores indicating worse function

^9^Clinical Dementia Rating (CDR) scale Sum of Boxes (SOB) score (addition of all subtotals), total score range: 0–18, with higher scores indicating more impairment

^10^Neuropsychiatric Inventory questionnaire (NPI) composite score, total score range 0–36, with higher scores indicating more severe neuropsychiatric symptoms

^11^Geriatric Depression Scale (GDS) Long Form scale, total score range: 0–30, higher scores (> = 14/30 concerning for depression) indicating screening positive for depression.

^12^Sources of diagnostic information included brain tissue pathology, cerebrospinal fluid (CSF) biomarkers of neuronal cell injury (i.e.14-3-3 Western blot, total-tau and neuron specific protein levels [45]), CSF RT-QuIC analysis [41], our internal review of the brain MRI(s), and prion protein gene (*PRNP*) analysis (done through the US National Prion Disease Pathology Surveillance Center, Cleveland, OH) [42]

^13^PRNP (prion protein gene) codon 129 genotypes of sCJD are MM: homozygous for methionine; MV: heterozygous for methionine and valine and VV: homozygous for valine.

^14^The one patient with inadequate quality MRI was positive for 14-3-3, total Tau (>4,000 pg/mL), RT-QuIC, and brain tissue pathology testing.

^15^The patient whose family refused autopsy had negative 14-3-3 and total Tau, but a positive MRI, and RT-QuIC, meeting UCSF, European 2009, and European 2017 probable sCJD criteria [15].

**Figure 1.**
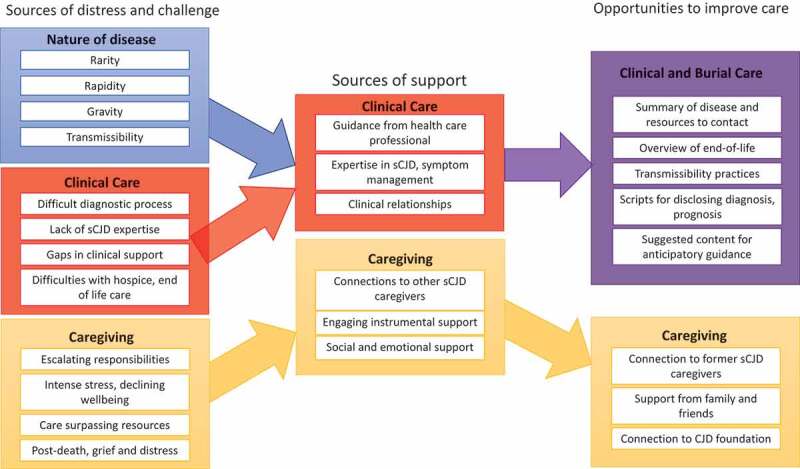
Summary of findings and implications for intervention targets.

Caregivers were interviewed a median of 22 months (range 11–39) after the death of the patient and had a median age of 59 (range 45–73; Table 1). Three-quarters were the patient’s spouse; half self-identified as female (50%); most had a college degree or post-graduate education (75%). Below we summarize themes within the challenges, supports, and recommendations shared by caregivers (Figure 1), providing examples in the text as well as in Tables 2–6.

**Table 2.** Sources of challenge and distress related to the nature of sCJD; quotes from interviews with bereaved caregivers (n = 12)

Theme	Exemplar quote for challenges related to the nature of sCJD
Rarity	
Lack of information, treatment or trials	‘it’s a lonely thing because unlike breast cancer, you know, everybody knows about it and everybody’s fighting for it but this, nobody knows about it and there’s not enough funding for it but it’s out there so it is a lonelier battle to fight than some of the other big diseases’ (c6)
Difficult to obtain diagnoses because of rarity	‘I came down for his appointment and we went to the emergency room and they said – they treated him for kind of like stroke symptoms and stuff and they said, “No, there’s nothing,” … they let him go and I wasn’t happy with it and I argued with the staff and I said, “Something’s wrong.” And they said, “Sorry, we can’t help you.” And I said, “Well I’d like for him to be admitted into the hospital … And I want a battery of tests run on him.” And they said, “There’s really nothing we can do, he’s passed everything, we don’t see anything wrong.” And I said, “Well look at his leg, look at him walk.” And they said, “Well, he says it’s because of a knee that he should have had reconstructed.” And I said, “Well, no … What do I need to do here?” And they said, “You have to get with your general practitioner and then he has to actually tell you that you need … [and] will get you into be able to see doctors in the … emergency room, it’ll get him past the emergency room.” And I said, “But I don’t think we can wait for this, he’s falling almost every day.” MSPKR: And so I stayed at my parents’ house for a few days until we could get this appointment with his general practitioner.’ (c7)
Difficult to find care from clinicians/facilities with sCJD experience	The hospital in [town] and the nursing home had never had anybody with that disease which is why most nursing homes were reluctant to take her.” (c1)
Rapidity	
Speed of functional decline	‘So, his symptoms right away were a memory thing and throwing up, and it was boom, boom, boom. His gait – he couldn’t walk anymore. He was upright, and all the sudden he was hunched over, and he’d have to hold onto walls and tables to walk.’ (c2)
Changes to family and social systems	‘So after that day when we were talking to him about “Where’s your trust?” it was kind of questionable, and we only had a certain window to sign that medical directive to maybe have a say in the hospice care or even the autopsy for [Health Center 2], because we had to make decisions like that, and who was going to be the lead person, and who was going to be in charge? And so, we only had a small window to sign that medical directive, and it never got signed, so [sister1] was kind of I guess in charge even though nobody ever stated it’ (c2) ‘it’s amazing how fast they change from that really capable hard-working amazing person to this person that’s not really there. I don’t think people realize how fast it changes.’ (c6)
Gravity	
Profound symptoms and functional losses	‘It wasn’t even safe. She was in a very small, tiny house that had huge drop-offs. She had fallen several times and nearly broken her leg because she was left alone, and so the more I observed and watched that I got to a point where I took it out of her hands, but by that time [patient] had quit walking. She wasn’t able to walk anymore, and her boyfriend knew that he couldn’t take care of her at that point, and he took her to the hospital because she was unable to walk, and at that point she remained in the hospital.’ (c1)
Difficult behavioural symptoms	‘when … I really saw how the auditory and visual hallucinations [patient] was having I knew we were in bigger trouble.’ (c5) ‘Periodically he would have these bouts of real anger still at the household help that they were stealing from him, that they had come and it started becoming clear to me that he was confused about where he was, that initially I said, “They’re not here, you are in [city1],” but he would be quite agitated.’ (c4)
Assured fatality	‘She began to get worse and before she lost her ability to communicate she told me how scared she was. And it was hard on me because there was nothing I could do for her, you feel helpless, there’s nothing you can do … It’s horrible way to die, to be trapped in your body like that, to be utterly trapped and she couldn’t do anything and I couldn’t do anything … by the time you do know what you’re dealing with it’s too late, it’s fatal, there’s nothing anybody could do’ (c1)
Transmissibility	
Impact on how people treated	‘Once they learned it was CJD, I noticed that their treatment of him changed. Meaning they were – I started noticing more precautions. They were taking a lot more precautions for themselves. Of course, there’s an ability for them to get some kind of contamination, but I told them, “Look, you would have to be, I think, blood or something of the sort.” But again, it goes to show some of the, let’s call it urban myths that may exist out there from the little of information that paramedics or doctors know of the disease. So, it’s almost like there’s an unwillingness of them to try to treat a patient that comes to the ER that has CJD.’ (c11)
Impact on facilities, burial options	‘We had a funeral home that said they wouldn’t even pick her body up. They would pick her up and they wouldn’t direct bury her because they didn’t know about it.’ (c6)

**Table 3.** Sources of challenge and distress related to clinical care

Theme	Exemplar quote related challenges of clinical care
Difficult Diagnostic Process	
Dismissive clinicians, protracted process	‘We had a horrible time with the neurologist and the hospitalist that they’re like, “She’s fine.” … I’m like, “She is not fine. This is not my mom.” … I always say we got kicked out of the hospital and then had to go back and spent two overnights in the ER because of the negligence of a couple of doctors that didn’t put any value into what I was saying about the changes in my mom.’ (c9)
Insensitive disclosure	‘I was upset and angry that if this was the diagnosis, the doctor wasn’t as sensitive. We’ve known him, he’s a family doctor for many years, and he’s been a friend of the family for many years, but how he gave us the news was sort of, “Look, this is what he has. This is a one-page. And he’s going to die from this. He’s going to die from this in about six months.” And he was very blunt, open, not a pulled you into an office. It was just very blunt.’ (c11)
Lack of prognostic estimate	‘All of this was very scary from the day they said “CJD,” because no one could tell me how long I had with my wife, and I would say “Well, am I gonna wake up one day and she’s gonna be gone? What am I dealing with here?” And nobody could answer that question, and that was the hardest part of all this, because I felt that death was imminent, and that’s a horrible place to be.’ (c12)
Lack of sCJD expertise, associated with inappropriate care	
Lack of expertise	‘We went to a top neurologist … and this particular doctor, she’s the top of her game at the hospital. And she was also at a loss. She completely was at a loss. And all she could do is really try to prescribe those drugs that are intended for people with Parkinson’s, or Alzheimer’s’ (c11)
Inappropriate care	‘he had to withstand that week of therapy at [health center1] because we didn’t know what he had, and at that point he should have just been resting, doing what he wanted. If he wanted to eat chips and beer I mean we were still at that point saying like, “should he be drinking this beer?”. It was like because we didn’t know, and I wish we were like. “whatever he wants just give it to him”’ (c2)
Gaps in Clinical Support	
Need for family caregivers even in facilities	‘having them come and telling me that, you know, I need to take care of myself and, you know, I’m the caregiver and but they couldn’t give me any times on when doctors would be there, when anybody would be there, so I had to sit there and wait for doctors. Like, and I waited actually for one doctor for four days’ (c9)
Abandonment	‘One thing that frustrated me was her neurologist. When the IVIG solution failed or when the infusion failed, I didn’t hear from her again. She didn’t call to check on her. I mean, she immediately dropped [patient] into hospice and wished us luck, and that kind of hurt. I had bigger things to do at that point, but I felt abandoned.’ (c12)
Difficulties with hospice and end of life care	
Difficulties accessing hospice despite prognosis	‘I remember when we contacted hospice and they came over, they evaluated, they said, “No, he’s not bad. He’s not bad enough,” and I was blown away at that and then – but it was, I want to say, 10 or 14 days later that he was bad enough’ (c7)
Hospice lack of expertise in sCJD	‘We had a hospice company coming in and I ended up … switching to a different hospice company because I was so frustrated. Every single time it was a different person that had no idea about her case, and they had absolutely no idea what she was going through. They had no idea what CJD was. And that was really, really frustrating. Because, you know, everybody wanted to know what were her benchmarks from yesterday and how is she doing? And it’s like, that’s not the way this works. <laughs> Whatever happened yesterday is out the window today, you know. It’s worse today and it’s going to be worse tomorrow and it’s going to be worse the next day. And it’s like nobody understood that. And even if by some chance somebody came back for a second day, they were surprised to see that she had declined. … And then we ended up switching hospice companies and quite honestly, the second one wasn’t much better, we still had the same problems with continuity of care and people just not knowledgeable about what they were dealing with.’ (c9)
Insufficient care	‘if the hospice nurse was not like so insufficient. Like the person, she was really great, but she was spread so thin that she was either running off to somebody who was ready to go, or she was coming from very long distances and could not come regularly.’ (c4) ‘The support for family, there’s nothing really there, and hospice does a good job for end of life, but they don’t know how to care for people that [aren’t] in pain at all’ (c7)
Discrepancy between what hospice recommended and caregiver is ready for	‘I did not want to give my dad morphine and the hospice nurse insisted, insisted, insisted. I guess the lead nurse or the boss per se visited our house and she pleaded with me and literally convinced me to do it because she kept talking to me on, “He is under so much pain.” … And I had kept my dad free of any morphine whatsoever … And I guess out of sympathy because of hospice, I gave them the go ahead for them to give him the morphine. When they gave him the morphine, needless to say, an hour later he passed away. I think the drug was just way too much in his delicate, frail state. So I think for me, ethically, that’s one of those things that I carry’ (c11)
Post-death activities	‘it took hospice, oh, God, two or three hours to get there [after she died]. And the person that they sent was just really not compassionate at all. She had her cellphone on speaker telling people …. And just very insensitive. And then we had to wait another several hours for her body to get picked up.’ (c9) ‘I felt like I had to make that and have them do that autopsy for various reasons, but I think that was handled very poorly, and, I mean, just after having lost my wife … and then going through that for many, many, many months longer than it should’ve taken was really, really difficult to deal.’ (c10)

**Table 4.** Sources of challenge and distress related to caregiving

Theme	Exemplar quote for caregiving challenges
Escalating responsibilities after symptom onset	
Assuming role as caregiver, advocate, decisionmaker	“They took him out of the house and loaded him up and they went to [Health Center 1], and he had pneumonia … and that was tough, because … there were decisions that needed to be made at that point … they go, ‘So with the DNR in place, are you just saying you want this to run its course?’ and I go, ‘Well, DNR is, what, Do Not Resuscitate, but he’s ali – like, he’s breathing. I mean, he’s alive.’ And they said, ‘Well, do you want us to treat this or not?’ and I said, ‘I don’t even know what you’re asking,’ and they said, ‘Well, if we don’t treat it, then he’ll, you know, we can release him to a facility with no medication and he’ll pass away from pneumonia,’ and I just wasn’t – I don’t know. I guess I wasn’t ready to even deal with that because, I mean, for me, a Do Not Resuscitate is like, I mean, you’re unconscious. I don’t know. I might not be astute enough to understand all those details, but basically they’re just saying, ‘Do you want us to let pneumonia kill your dad?’ ’ (c7)
Lifestyle changes	‘I decided I would sleep in the bedroom that was just right across the hall and leave the door open and it was like if you need something just call me, and I had the sense to put a baby monitor in there, and so I could hear him getting up, trying to get up at night to go to the bathroom, and I’d come in and I’d go [husband, patient] you can’t do that … so I would have to jump out of bed the minute I heard any rustling, and so even be able to get a good night’s sleep or sleep deeply at that point in time it was just really hard.’(c3)
Helping with ADLs	‘I actually wasn’t able to spend quality time with my dad because I was cooking and cleaning … He would have that thing and he’d pee in it and then he would go to grab it and then he’d knock it all over the place, and so then I would have to sit there and clean up pee, you know, for an hour, and then I’d, you know, have to go and make him lunch’ (c7)
Intensifying stress and declining wellbeing	
Intensifying stress	‘So, we had various family come through and we were caring for him ourselves, it was frankly becoming quite difficult, I had taken on too much, I would sleep in the same room, I’d put him in the master bedroom, my wife moved to the guest bedroom, I would sleep in the master bedroom waking up whenever he would get up and so on.’ (c4) ‘We had to sort of be gatekeepers to say, “You can come,” or, “You can’t come.” And it’s really interesting how everybody … “Well, I’m her friend I need to see her.” “I know you’re her friend but,” and it was very tiring, it was very difficult to say no to people’ (c5)
Declining wellbeing	‘Oh god. It was really awful. It was stressful.’ (c3) ‘I literally lost 20 pounds taking care of my wife.’ (c12) ‘I got to the point where the stress sent me to the emergency room four times. It did. It did … What they told me is, “You’re not having a heart attack. … But it’s not to say that the stress will not give you a heart attack.”’ (c11)
Care needs surpassing available resources	
Family caregiver help insufficient at some point	‘ … would say that that was one of the toughest things, that he was always – He would be shaking and fumbling for the urinal and then he’d miss and then you’d want to assist him. And, you know, but he’s doing his own thing, you know, and so that’s kind of tough for, you know, a daughter or, you know, a family member to have to do that. That’s why it’s good to have a nurse or an assistant. But if you can’t have that, then what do you do, so?’ (c2) ‘I was able to do that for maybe a couple of weeks and then I finally brought somebody in to help me because I couldn’t do it anymore on my own. I was too worried about him falling, me falling, and the whole nine yards.’ (c3) ‘So, I feel like just towards the end, and I’ll be frank, you become ambivalent, you want him to pass, you don’t want to <participant is crying> prolong it, you want him to be comfortable yet it’s a very difficult phase, so I made that decision to take him to … the long term care’ (c4)
Limited avenues for other support	‘I even had hired help like nurses. We lost one nurse. Another one came in, she was a little bit more negligent. She allowed an infection to develop in his dentures. Like, things that you overlook, I should have known, okay, you can’t – If this nurse was not changing out his dentures, might as well leave them out so he doesn’t build up food within his dentures. He developed an infection and I don’t know if the infection was what killed him, but within two or three days later, he ended up passing away.’ (c11) ‘Or the caregivers are cleaning him in a way that there’s too much moisture and he’s not dry enough. So, I had to kind of watch for those things because her examination of him when she came was limited by the time she could budget which I’m sure limited by cost considerations.’ (c4)
Challenges post-death	
Final arrangements for decedent	‘We had told the hospice people … because it is a very rare disease that you need an autopsy to confirm the cause of death or the disease, we want to make sure whatever’s written on the death certificate to be correct … you want to make sure because it’s like important that is it mad cow, is it sporadic, is it genetic. So thank god we caught it in … we called the person in charge of the hospice that deals with death certificates and the mortuary and they changed it because it hadn’t yet gone through the county yet, and they changed it to I think something encephalitis, and then when the autopsy was finished months later and confirmed that it was sporadic CJD then the death certificate was finalized as sporadic CJD.’ (c2)
Ongoing grief and distress	‘I mean talking about it now again makes me want to cry because of what I saw my sister go through but anything I went through is not nearly as bad as what she suffered. I still think about it every day what she went through, and it’s been a year and a half since she’s passed.’ (c1) ‘By the way I read about it because again even though my dad had sporadic CJD it still makes me think why did he get it, like just naming it sporadic doesn’t make me say oh, okay, he just got it kind of because, like there’s no reason … When you lose your parent you kind of want to know why.’ (c7)

**Table 5.** Sources of support and amelioration related to clinical care

Theme	Exemplar quote for clinical supports
Guidance from clinicians/health care professionals/researchers	
Referrals	‘I’ve always wanted to thank him and write him a note, because he got us on the right path’ (c2)
Diagnosis and prognostic estimates	‘I said “Okay, so, Doc, and the question – you got to know it’s coming. How much time do I have?” and he paused for a moment, and he said “Well, based on what we can see I think you have another six to nine months” – he said, “six to 12 months with your wife,” which was a relief.’ (c12)
Confirming what caregivers learned elsewhere (online)	‘I did talk at length with a neurologist there … I wanted to confirm that it was fatal, that there was no cure and they told me. I wanted to know what the lifetime span was. And so the questions that I asked him was because I wanted to confirm what I had been studying to make sure that … there was not misinformation that I was reading’ (c1) ‘With [the attending physician] we spent about 20 minutes. But with the team there, personally, I learned a lot. I learned what to expect. It complemented a lot of the research that I had. It also allowed me to – after we left to … research on my own. And better manage the situation.’(c11)
What to expect over time	‘He said – now remember, we saw a picture of her MRI. And if you’ve never even seen an MRI of a brain he pointed out and he said, “This part doesn’t function anymore. This part doesn’t function anymore. This part. And it will soon become her entire brain that no longer functions,” because this disease, for lack of better words, eats it away which is a pretty hard visual. And so she will go from being able to talk and request and feed herself to not being able to feed herself, to the point of not being able to eat’ (c5)
When to seek more help	‘I found a social worker here locally that – she was very instrumental in helping me through the hospice question. And she said – and, again, we agreed that, okay, when this happens and this happens, you need to call me …. And then her and I had worked out, “[interviewee], here’s what you need to do. When you can’t feed her anymore and she’s been incontinent,” she said, “you need to think about engaging hospice.”’ (c12)
How to identify imminent death	‘The hospice did because I told them … I want to know what the signs are when she’s down to a matter of days, and they told me. They said she won’t be able to eat, she won’t be able to drink at all, and her sleeping ability will rapidly change, which it did.’ (C1)
Expertise in sCJD and symptom management	
Reassurance of expertise	‘ … although the ultimate diagnosis was not what we wanted, but we were with people that knew what they were dealing with and weren’t dismissive and they did a much better job caring for our mom and for us.’ (c9)
Advice on how to manage symptoms, adjust medications	‘I got really good help from [the attending physician] himself, the team. I would just email him and say this is what is happening, and they would respond. Even sometimes they recommended change in medication in consultation obviously with the psych doctors here, and at some point they even recommended that most of the medication that he’s taking, just stop those medications, like he doesn’t need cholesterol control anymore.’ (c4) ‘It was not only guidance, it was also if you see these symptoms, try to calm him this way. So, for example, we were told about music. We were told about light. We were told cloudy days he’s going to go bad. If it’s raining outside with the barometric pressure, he’s going to be affected. But on a sunny day, take him outside. It was like medicine. You take him outside on a sunny day and he was alive. You would see him attentive.’ (c11)
Confirmation of variant type	‘Well first thing it was I just burst into tears when I found out it was spontaneous and it wasn’t genetic because we were again already under so much stress, and then to find out at least it was not genetic was huge.’(c3)
Supportive clinical relationships	
Sensitive, responsive, proactive communication at every stage	‘So while they’re doing the exams, I’m asking questions. As I’m asking questions, the people doing the exam were very transparent. And that, to me, was very, very valuable … It’s not like they were trying to withhold information. Any question I asked, “Why is this being done? What’s the purpose of this?” … Everything was being answered.’ (c11) “They [hospice clinicians] told us they were going to do it like a dementia patient because she exhibited lots of signs of dementia. And I don’t know if they were really that educated on the actual Creutzfeldt–Jakob but they were amazing for our family, I think we were learning together, it was more like, ‘We’re all in this together at this point.’ And they would ask me, ‘You’ve been through this with your mom for the last year and a half, two years, whatever, so we are really open to what you think is best.’ (c6)
Prompting caregiver self-care	‘I remember at one point in time she had said, “Are you talking to anybody like, you know, a counselor or anything?” And I had said, “You know, I hadn’t even thought of it because right now it’s just–” Like, you know when you’re caring for someone it’s’ a job and you’re, you just want to do a good job and you don’t want to leave any stone unturned.’ (c7) ‘a doctor in one of the emergency rooms and he was able to give me – He sat with me. He started to explore, “Okay, you’ve been here now twice to this emergency room. You’ve been to other emergency rooms.” … He was trying to understand based on my records why was I there. And when I started telling him the story he just said, “Okay. Your issue with your dad is trust.” And that’s when he said, “You’ve got to bring in a team. There is such a thing called hospice.” But at the same time, he said, “Look, just hire people if you can afford it.”’ (c11)

**Table 6.** Sources of support and amelioration related to caregiving

Theme	Exemplar quotes for caregiving supports
Connections to other sCJD caregivers	
Reading online stories or seeing videos of experience with sCJD	‘I came across an article that somebody posted of her husband who had that disease … It helped me in knowing as I was watching my own sister the same things were happening. I can’t explain it. It’s just somebody’s actual experience makes a difference.’ (c1) ‘Where I felt like what really helped me the most was finding people that have been through this, so YouTube videos … was great.’ (c7) ‘I think it’s CJDfoundation.org or something like that. Because they’re the ones that I finally registered my mom with. And then that’s when I started finding people like me that I emailed and like, “Oh, hey, I saw your story. This is my story.” And so, I didn’t know that until I started rifling through stuff.’ (c6) ‘I interviewed doctors. I interviewed researchers. I interviewed people – I did cold calling. … I mean, I was phone banking from six in the morning till twelve midnight trying to get a person on the phone. … But there are others that were very forthcoming, and compassionate and sensitive … I also was able to identify and find attorneys … that actually had their parents with CJD! So, I was able to develop my own network of support!’ (c11)
Engaging instrumental support	
Help with legal and financial concerns	‘So, my brother was a lawyer, so that really, really helped. He helped me get trusts set up … A friend of mine helped with taxes. Yeah, so I was very, very concerned financially. Once he [the patient] passed he had life insurance which was a life saver literally for us financially.’ (c3) ‘There was another thing went out that people were worried about us financially, so people were sending us money, and then the people we lived with, my best friend, they totally turned their lives upside down for us and could not have been more giving’ (c3)
Documenting end-of-life planning or wishes	‘I showed him the advance healthcare directive that my wife and I have written … and I said, “We wish to be kept comfortable but not prolonged and we are very young and we have written this,” and he [father, patient] read all that and agreed that that was the right way to go. So yes, he had agreed and signed so he was still cognizant enough of what he was signing, so fortunately that was already there.’ (c4) ‘my brother sent me this book that helped with planning what you want. So [husband, patient] was able to fill out some of the information in terms of what he would or would not want, and so that really helped’ (c3).
Hiring paid caregivers	‘We had a caretaker that was going to come in now and then to help, you know, bathe him, because we really don’t know, you know, how to bathe a patient like this.’ (c2)
Transferring patient from home to facility-based care	“ … where I worked I have someone that’s a mentor to me in life and in general and business and he had said, ‘You know, maybe with this care facility you guys could get to, you know, more of the good part of living instead of being overwhelmed by, you know, all of the hard work and those details in the bathroom. And so that’s exactly what happened’ (c7) ‘ … the staff at the home where she was. I felt comfortable leaving at night and going home and getting a good night’s sleep. And so that I could come back and do it all over again the next day.’ (c9)
Engaging hospice	‘ … then they just did everything and ordered the bed was in our home within hours kind of thing. You know, they told us you should get the … pad for the bed. I mean, it was just wonderful, that whole part. And we ordered the medications, Lorazepam and morphine to have there when he got home so that if you could – needed to start distributing it. And it was just boom, boom, boom.’ (c2)
Finding a funeral home to meet sCJD needs	‘The funeral home here, they did the autopsy and took her brain and they took care of it and it wasn’t a problem like we kept worrying that maybe it was going to be and because of all the considerations for the disease. But it ended up not being a problem at all and it got handled’ (c9)
Social and emotional support	
From friends and family members	‘almost every day people were coming in and bringing him milkshakes and Frappuccino’s and cookies, and just spending time with him ….the guys that he played tennis with would come over and … they’d roll him over [in his wheelchair] so he could watch a tennis match.’ (c3) ‘We had a lot of the people that stayed overnight and helped out, so myself and my son, we could get some rest.’ (c10)
Strategies to maintain connection between caregiver and person with sCJD	“I would raise his bed, I would feed him by hand a little bit, I would keep him hydrated, give him water. … and again culturally to us that is – we feel a lot of pride and comfortable in being able to take care of somebody to feed them by hand. “ (c4) ‘I think the best medicine for me was taking care of her. I saw her in a different light. I saw her as a very humble human being that needed me. And I would tell them you have every right to be angry. It’s frustrating. And it is heart wrenching that your loved one is going to be taken from you so quickly and there’s nothing you can do.’ (c6)
Promoters of caregiver emotional resilience	‘we’re a very religious family … I really believe there’s something after this life and so that was integral in me staying grounded.’ (c6) ‘They encouraged me to make sure that I take care of myself. So – and I did. … I had a bike down here. my sister-in-law would spell me for, you know, an afternoon so I could go ride my bike.’ (c12)

This figure summarizes qualitative analysis of interview data from former caregivers of patients who died from sporadic Creutzfeldt-Jakob disease (sCJD). We identified 3 major categories of challenges and distress – nature of disease (blue box), clinical care (red box), caregiving (gold box). We also identified 2 major categories of support related to clinical care (purple box, reflecting that both the nature of the disease and clinical care are at play) and caregiving (gold box). Opportunities to improve care are organized into clinical (purple) and caregiving (gold) categories, and reflect what caregivers said helped, what they recommended or wished for, and researcher recommendations on how to address challenges as reported in the discussion of the manuscript. Each box summarizes the major themes within each category of challenges, supports, or opportunities.

### Sources of challenge and distress

We identified 3 major categories of challenges and distress – nature of disease, clinical care, caregiving.

#### Nature of sCJD

Distinguishing features of sCJD – its rarity, rapidity, potential transmissibility, and gravity – consistently appeared across respondents’ narratives as independent challenges and as compounding factors (Table 2). sCJD’s *rarity* meant caregivers encountered a lack of information, expertise, treatment options, and trials, causing protracted diagnostic journeys and inappropriate care.

Challenges related to the *rapidity* of the disease included the pace of functional decline: ‘*you suddenly find that at every plateau you upgrade your skills to deal with that plateau and when they fall off that plateau with another function going, it is like you’re not expecting it*’ (c4). One caregiver described their father using a cane for only 10 days before requiring a walker, and then moving to a wheelchair in two months (c7). Caregivers also described correspondingly rapid changes to family and social systems, such as their need to quickly take on legal and medical decision-making responsibilities (c2).

Challenges related to the *gravity* of the disease included loss of almost all ADLs, difficult behavioural symptoms (e.g., hallucinations, panic/fear, wandering: ‘*all of a sudden she got really hallucinating and being really afraid of things and it just started snowballing from there’* (c6)) and assured fatality.

A few caregivers described challenges related to *transmissibility*: the potential that prions, the misfolded proteins causing sCJD, could be transmitted to others. Some believed that their loved one was treated differently, or rejected from facilities, because of transmissibility concerns. One caregiver said that a funeral director said: ‘*Oh, you have Creutzfeldt, well we’ll come get her but we’re going to direct bury her because we can’t embalm her*’ (c6), contrary to the family’s wishes and accepted medical practice [21–23].

#### Clinical care

Caregivers reported many challenges and sources of distress related to clinical care, clinicians, or health systems (Table 3). Almost all experienced extensive challenges in obtaining a diagnosis, often attributed sCJD’s rarity. Caregivers evinced frustration at doctors’ dismissal of early symptoms and at making multiple clinic trips in pursuit of a diagnosis. Clinicians’ ack of sensitivity in disclosing this terminal diagnosis caused distress: *‘it didn’t seem like there was a lot of concern on their end’* (c10). The lack of clear prognostic information to inform planning also was a common problem.

Caregivers conveyed distress at many clinicians’ lack of expertise in sCJD: ‘*I don’t think we ever saw anybody [prior to UCSF] … who had any idea about what this disease was or how it progressed or how to deal with someone that had it*’ (c10). Caregivers suggested this led to unsuitable care, including inappropriate or harmful medication or care plans ill-suited to an RPD.

Gaps in clinical support were a challenge. Particularly in hospitals or facilities, caregivers felt they needed someone present to help or advocate for the patient: ‘*We were there 24/7 because … [staff] probably wouldn’t have come around or known that he wet the bed’* (c2). Caregivers were extremely distressed if clinicians no longer helped them (‘abandoned’ them; c12) after suspected diagnosis or enrolling patients in hospice.

Caregivers described distress at end-of-life care and post-death support. Hospice sometimes delayed enrolment because they did not understand how rapid the decline would be in sCJD. Though predominantly perceived as helpful, as detailed below, hospice care was also insufficient: ‘*[The hospice nurse] was really great, but she was spread so thin … and could not come regularly*’ (c4). Hospice staff did not always know or learn about sCJD, sometimes misinterpreting symptoms, such as treating myoclonus, pyramidal or extrapyramidal symptoms as pain and managing the patient more like ‘*somebody with cancer*’ (c7). Challenges did not end upon the patient’s death – delays and administrative hurdles with body handling, funeral home and autopsy arrangements, pathological and genetic results were common issues.

#### Caregiving

Caregiving challenges spanned the disease course and post-death (Table 4). Respondents described difficulty accepting the diagnosis or prognosis of sCJD, which sometimes interfered with fulfiling caregiving or decision-making roles. Caregivers took on new roles as advocates and decision-makers: *‘I found it very difficult having no knowledge of this and trying to get educated on it in a short period of time’* (c5). Caregiving intensified quickly, often involving major lifestyle changes. Several respondents took leaves from work and/or moved in with their loved one. ADL help was hard on both parties, from patients not accepting help, to the new caregiving role preventing them to simply ‘be’ with their loved one.

Caregivers experienced intense stress and sacrificed their own wellbeing to care for the person with sCJD ‘*I really could not believe that … I didn’t have either a nervous breakdown, a heart attack or something, because the level of stress*’ (c2). Respondents expressed sadness or anger at the many losses experienced by the patient and worried about their suffering.

Caregivers struggled as care needs of the patient surpassed available resources, eventually recognizing their own limits: ‘*I finally brought somebody in to help me because I couldn’t do it anymore on my own*’ (c3). Obtaining professional caregiving was challenging due to the high cost or limited availability, with some facilities unwilling to care for someone with CJD. Caregivers had limited means for other support: ‘*I wasn’t able to afford on a cash basis somebody to come twice a day seven days a week to take care of his incontinence and so I had to do it myself still in spite of hospice coming … It’s not comprehensive enough*’ (c4).

Caregivers experienced ongoing grief, fatigue, and distress after the patient died: ‘*it took me a good year to kind of recover from the emotional and physical toll that it took on me’* (c4). One compared her experience to post-traumatic stress disorder, noting that aspects of it still ‘haunted’ her (c9). Reflecting on the caregiving experience, many expressed sadness or cried during the interview.

### Sources of support and amelioration

We identified 2 major categories of support – clinical care and caregiving.

#### Clinical care

Despite challenges, many respondents also experienced clinicians and clinical care as sources of support (Table 5). Caregivers appreciated clinician guidance about diagnosis, prognosis, or referrals. Anticipatory guidance was particularly helpful: to understand the patient’s disease trajectory, *‘[the doctor] told me to focus on the rate of change of various phases, … [if] it’s accelerating or he’s moving to another function loss that is your sign that he’s moving forward. But if he stabilizes somehow … that may not be sign that’s [he’s] ready to go. That really helped me*’ (c4). Guidance about when to seek additional help and the expected disease course, including how to recognize signs of imminent death, helped caregivers feel prepared, take breaks, and feel less guilt.

Caregivers valued clinicians’ expertise in sCJD, particularly early in the disease journey, when detailed explanations of the disease were helpful. Respondents appreciated expert management of medications and advice about behavioural adaptations to manage sCJD symptoms. Confirming the CJD as sporadic (not genetic) was a source of relief for nearly all.

Sensitive and supportive relationships with clinicians and researchers stood out, such as when clinicians responded quickly and thoroughly: ‘*I could call her or text her anytime and she would be answering questions for me*’ (c3). Clinicians and others with prion expertise helped caregivers prioritize self-care: ‘*[Dr. III] himself …. advised me strongly to back off from [caregiving], that I would potentially cause harm to myself that could be damaging. So I really appreciated that advice*’ (c4).

All caregivers reported engaging hospice, sometimes before the diagnosis of CJD. Though some caregivers expressed frustrations with hospice clinicians or the care model (especially lack of continuity), most found hospice helpful for providing hands-on ADL support, breaks, and comfort. They benefited from fast implementation and hospice staff’s expertise in recognizing imminent death and appreciated when hospice made effort to learn about sCJD and inquire about respondents’ knowledge as sCJD caregivers.

#### Caregiving

Finally, respondents identified sources that facilitated being a caregiver (Table 6). They emphasized the benefit of reading or hearing stories from, or connecting with other sCJD caregivers, via YouTube, Facebook, and the CJD Foundation: ‘*all you want to do is talk to people that have been through it. Because you don’t know what to expect’* (c7).

Caregivers benefited from instrumental support (e.g. paid caregivers or facilities relieved caregiving burden): ‘*I did all the heavy lifting at our house … When we transferred to the care facility, I felt lighter*’ (c7). Friends or family also provided ADL, legal or financial help, such as documenting preferences or decision-makers while the patient was still able to make decisions. Many narratives indicated that socioeconomic resources were essential, such as being able to pay for caregiving, having access to state-funded care, and/or having jobs that permitted reduced schedules and lengthy leaves of absence.

Social and emotional support was beneficial. Much of this came from friends and family, e.g., keeping the patient company or reminding (and helping) the caregiver to take a break: ‘*There wasn’t anything to do except support her and everybody was ready, willing and able to sign up*’ (c5). Some caregivers benefitted resilience-bolstering activities, such as religious practice or exercise. Others found comfort in maintaining close connection to the person with sCJD; one caregiver did ‘spa days’ for his wife after she was bedbound (c12).

### Caregiver recommendations or wishes

Caregivers also identified items that they thought would have been helpful to them or future sCJD caregivers. Though hypothetical, these insights may be useful for intervention development.

Regarding clinical care, some caregivers felt earlier accurate diagnosis and familiarity with sCJD among clinicians, hospice staff, and funeral home directors would have made the experience less difficult. Some recommended that clinics provide resources about expected symptoms and prognosis, what to take care of (e.g., advance care planning), resources (e.g., CJD Foundation, support groups, local hospice organizations), and contact information for expert advice about sCJD management to give facilities, hospices, and funeral homes.

Regarding caregiving, some respondents thought they would have benefited from more self-care and time connecting with the patient. When asked for recommendations for future sCJD caregivers, respondents echoed these themes: spend more meaningful time with the patient, have more patience with themselves and the patient, connect with other sCJD caregivers, and engage hospice care.

## Discussion

This novel study provides an expanded understanding of challenges experienced by caregivers and persons with sCJD and identifies opportunities for improvement. Challenges primarily related to clinical care and caregiving and were exacerbated by the unique nature of sCJD. To our knowledge, this is the first in-depth description of palliative care needs of persons with sCJD. Ford et al. (2018) previously studied caregivers’ struggles to manage symptoms of patients with sCJD and found the most problematic to be mobility and coordination, mood and behaviour, personal care and continence, eating and swallowing, communication, and cognition and memory [20 – 23, 7]. Caregivers in our study voiced similar challenges with symptoms, and described broader sources of distress and challenges. Caregivers framed changes in patient function within the larger context of major losses and changes to relationships, life plans, and family roles. We additionally asked about supports to identify factors that ameliorated caregivers’ difficulties. Supports were often the inverse of challenges, such as sensitive versus insensitive disclosure of diagnosis and prognosis.

Data on sources of distress and support in sCJD facilitates the development of neuropalliative tools and interventions. Table 7 demonstrates how palliative care approaches might be integrated into neurology practice for sCJD and slower-progressing dementia syndromes. For example, neurology trainees can be taught to use serious illness communication strategies [24] for sensitively disclosing a diagnosis of sCJD and asking if patients and caregivers want prognostic information or anticipatory guidance at this time [25,26]. Findings from this study can facilitate improving sCJD-specific care among hospice and palliative care clinicians. Neuropalliative-infused interventions for improving sCJD care will need to be refined with interdisciplinary multi-stakeholder input and tested for utility and effectiveness.

**Table 7.** Neuropalliative care intervention targets, and solutions, in sCJD

Target	Justification from study data	Solutions: Tools/resources
For sCJD experts and other clinicians		
For clinicians not expert in sCJD	Challenge of lack of sCJD expertise	Overview of disease, prognosis, and timeline Information about diagnostic criteria for and management of sCJD including brain MRI, clinical presentation, and positive RT-QuIC [17]: www.cjdfoundation.org (USA); https://cjdisa.org (international); https://memory.ucsf.edu/dementia/rapidly-progressive-dementias; https://www.cjd.ed.ac.uk/ (UK); List of common symptoms and recommended management strategies [17] (https://memory.ucsf.edu/dementia/rapidly-progressive-dementias) List of medications to consider using or avoiding [17] (https://memory.ucsf.edu/dementia/rapidly-progressive-dementias)
Reminders for sensitive disclosure of serious diagnoses	Challenge of the diagnostic journey	Serious illness communication strategies [24] such as NURSE, [25,26] SPIKES, [46] and/or the Serious Illness Guide [47]. Key principles including whether to disclose at initial or dedicated follow-up appointment, allowing patient/caregiver to choose who to be present, having enough time to disclose diagnosis and answer questions, and providing written materials
Prognostic information, caregiver training, and anticipatory guidance	Requests for summary of disease, anticipatory guidance	Information can be aggregated from this and future studies; should include common safety concerns (driving, falls), advance care planning, [48] how to provide assistance with activities of daily living, how to recognize need for additional help (e.g. paid care) and nearness of end-of-life (see prion disease resources below)
Holistic support of patient and caregiver	Experience of support when clinicians ask about caregiver wellbeing, help facilitate planning for end-of-life care and caregiver support	Regular inquiries into caregiver wellbeing and self-care strategies Normalize speed of progression, potential loneliness of rare disease Ask about interest in, progress towards, documenting surrogate decision maker and patient/family preferences (end-of-life) Manage expectations about palliative, hospice and/or end-of-life care (e.g., need for additional paid care)
Additional emotional supports	Receiving emotional support from clinical teams	Having social worker on care team, genetic counsellor if genetic testing ordered, and/ or having support infrastructure, such as the Care Ecosystem, [49–52] available. Consider referring to www.cjdfoundation.org for webinars and 24-hour support line (USA).
For caregivers/patients		Paper and/or digital compilation of resources
List of potential preparations and decision support	Recommendation to provide lists and resources to support planning	Advance care planning includes naming and documenting surrogate decision maker, signing advance directive and medical durable power of attorney, completing a POLST form (https://polst.org/), and deciding whether to enrol in a brain donation (autopsy) programme. Financial and legal decisions include signing financial power of attorney, transitioning accounts/billing, will/trusts. Burial preparations include choosing funeral homes, discussing memorial arrangements. Advanced care planning web-based resources [53] include PREPARE [54,55], SAME Page [56]; Think Ahead [57]; or ENVINCE [58] advance care planning videos for people living with dementia.
Local service resources	Recommendation to provide list of local services	Names and contact information for local home health agencies, nursing facilities, hospices, therapists, social workers, genetic counsellors, support groups
Some prion disease resources	Recommendation to connect to reputable sCJD experts	Websites such as www.cjdfoundation.org (USA), https://cjdisa.org (international), https://memory.ucsf.edu/dementia/rapidly-progressive-dementias, https://www.cjd.ed.ac.uk/ (UK), https://www.cdc.gov/prions/index.html
Methods for learning about experiences with sCJD	Experience of other sCJD caregiver stories as source of support	Links to website(s) aggregating written, video stories, methods to contact other sCJD caregivers, such as https://cjdfoundation.org/
Recommendations for engaging family/friend supports	Experienced help and recommendations to accept help	Lists of examples of ways that other people living with sCJD and caregivers have engaged or accepted help from family and friends (e.g. extracted from this and other studies). Some helpful sites: https://www.caringbridge.org/, https://lotsahelpinghands.com/
Sheet with information about prion diseases to give other clinicians, hospice staff, funeral homes	Challenge with hospice and funeral homes lack of expertise with prion disease	Many copies of written 1-2-page summaries that caregivers can give out re: basic information, managing transmissibility concerns, phone number of the NPDPSC Autopsy programme at Case Western Reserve University (www.cjdsurveillance.com). Websites for funeral homes: https://case.edu/medicine/pathology/sites/case.edu.pathology/files/2020-10/Prion%20Diseases%20-%20Autopsy.pdf, https://cjdfoundation.org/funeral-professionals, & https://www.cdc.gov/prions/cjd/funeral-directors.html

Evidence regarding neuropalliative care needs in sCJD may be applicable to other rare and rapidly progressive diseases with no cure, as well as longer-course neurodegenerative diseases. A recent systematic review of factors influencing the provision of palliative care to persons with advanced dementia report similar problems: difficulty managing symptoms, lack of continuity of care, and lack of clinician skill in palliative care (such as sensitive disclosure of information or providing anticipatory guidance) [27]. A systematic review of integration of palliative care into dementia management highlights the importance of discussing disease trajectory and expectations and challenges from suboptimal symptom and medication management [28]. These challenges appeared in our study as well. We are adapting the analytic approach of this sCJD study to our parallel efforts to identify neuropalliative intervention targets for longer-course dementia syndromes [29–31].

Limitations of the study include a relatively small sample at one institution, albeit one that recruits study participants nationally (and even globally) for this rare disease. Demographics of participating caregivers suggest that they are well-resourced. Caregivers with fewer resources may encounter more, or more severe, challenges than documented here. Future research should engage larger, more socioeconomically- and globally-diverse populations, and other RPDs that may raise different caregiving challenges. Nevertheless, these novel findings provide foundational data for further research and intervention development.

In summary, this study drew on palliative care frameworks and mixed methods to yield a comprehensive description of challenges, supports, and opportunities to improve care for people with sCJD and their caregivers. Though sCJD is rare and rapidly progressing, the themes uncovered provide a framework for ongoing efforts to improve neuropalliative care for dementia care more broadly.

## Methods:

*Design*: We conducted an exploratory mixed methods study [32] to capture in-depth information about challenges and sources of support among persons with sCJD and their former caregivers. This study drew from interviews with former caregivers of people who died from sCJD and research chart data about the person with sCJD. It was approved by the University of California San Francisco (UCSF) Institutional Review Board and comports with the Consolidated Criteria For Reporting Qualitative Studies (COREQ; Appendix) [33].

*Participants and setting*: We identified caregivers from the UCSF Memory and Ageing Center (MAC) RPD research programme database, which includes extensive information on individuals who consented to the ongoing use of their data from medical records and/or from research records through an IRB-approved study of RPDs [34]. We purposively sampled caregivers of persons who died with sCJD at least 3 months but no more than 3 years previously to capture variation in degree of interaction with the UCSF MAC RPD research team and to capture variation in clinical presentation through sCJD molecular classification [19,35]. Of 23 candidate caregivers approached before recruitment closed due to COVID-19, 12 agreed to participate.

*Data collection*: Caregiver interview domains focused on key experiences along the patient’s disease trajectory; caregiver activities and quality of life; challenges and sources of distress; and things that did or could have helped them to care for the person with sCJD; and a demographic survey (Appendix). Phone-only interviews were conducted from September 2019 through March 2020 (median 88 minutes, range 41–161). Caregivers provided written consent and agreed to digital recording. Recordings were professionally transcribed.

We extracted demographic and clinical data on patients linked to recruited caregivers from the UCSF MAC RPD and UCSF MAC general research (‘LAVA’) databases. For patients who participated in a 2-day outpatient research visit, data included assessments of cognition [36] [37],, neuropsychiatric symptoms [38],, function [39] [40],, and disease characteristics (Table 1). For inpatients who were only seen in the UCSF clinical wards (and did not participate in the more extensive 2-day research visit), more limited data was extracted from their EPIC electronic health record and (if available) RPD and LAVA databases. Sources of diagnostic information included brain tissue pathology, cerebrospinal fluid (CSF) biomarkers [41], brain MRI summary, and prion protein gene (*PRNP*) analysis [42] (Table 1).

*Data management and analysis*: Patient data were summarized using descriptive statistics. Caregiver data were summarized in structured case summaries that included emergent themes and interviewer reflections. We iteratively reviewed chart data, case summaries, and transcript excerpts throughout data collection to refine analytic approaches and identify preliminary themes.

We employed both deductive and inductive coding to identify themes. Deductive codes reflected concepts from the interview framework: challenges or sources of distress, sources of help, and caregiver recommendations for improvements. Inductive codes reflected meaningful concepts emerging from the interviews (e.g., disease rarity, rapidity, transmissibility). Three authors (KLH, SBG and CSR) iteratively refined codes by double-coding and discussing discrepancies until agreement had been met; KLH applied the updated codebook to all transcripts. Analysis was guided by the constant comparative method [43], which uses iterative comparisons within and between analytic cases. The team maintained an audit trail of methodological and analytic decisions.

## Supplementary Material

Supplemental Material
