# Supplementary material for: Developing neuropalliative care for sporadic Creutzfeldt-Jakob Disease
Source: Prion. 2022 Mar 3;16(1):23–39. doi: 10.1080/19336896.2022.2043077 (PMC8896185; doi:10.1080/19336896.2022.2043077)
Supplement: Supplemental Material [file KPRN_A_2043077_SM3759.docx]

**Appendices**

**Appendix table 1:** Consolidated criteria for reporting qualitative studies (COREQ): 32-item checklist – summarizes methods for interviews with caregivers only

| **No** | **Item** | **Guide questions/description** |
| --- | --- | --- |
| **Domain 1: Research team and reflexivity** | | |
| Personal Characteristics | | |
| 1. | Interviewer/facilitator | PhD-level social scientist |
| 2. | Credentials | PhD |
| 3. | Occupation | Research sociologist |
| 4. | Gender | Woman |
| 5. | Experience and training | Doctoral-level training in sociology. Intensive expertise with collecting and analyzing in-depth interview data. |
| Relationship with participants | | |
| 6. | Relationship established | The relationship commenced with study recruitment and lasted for the duration of the interview. |
| 7. | Participant knowledge of the interviewer | Participants understood the interviewer’s and study goals: To learn about needs and experiences of persons with sporadic Creutzfeldt-Jakob disease (sCJD) & their families, hopefully to inform new or enhanced services at the specialty dementia clinic center. |
| 8. | Interviewer characteristics | Interviewers identified and reported no personal characteristics that would contribute to bias or interests beyond their interest to contribute to scientific knowledge. |
| **Domain 2: study design** | | |
| Theoretical framework | | |
| 9. | Methodological orientation and theory | The study draws on implementation science, with respect to trying to understand potential user needs and wishes to inform future interventions.  The project also is based in subtle/critical realism, acknowledging both subjectivity in describing reality, the socially constructed nature of reality, and also the existence of a reality that can be measured through the thoughtful collection of data and triangulation in analysis.  Study design was also influenced by the interprofessional, international study team, which included people with training in public health, health policy, ethics, sociology, anthropology, behavioral neurology, neuroscience, geriatrics, hospice, and palliative medicine. |
| Participant selection | | |
| 10. | Sampling | Potential participants were identified based on data captured on patients with sCJD in the University of California San Francisco (UCSF) Rapidly Progressive Dementia (RPD) Study Database. Caregiver participants were recruited from among those who were listed as contacts for patients in the CJD database. We aimed to capture variation in degree of interaction with the UCSF RPD research team (e.g., patient who was participant in 2-day research study vs. inpatient clinical care) and to capture variation in clinical presentation through sCJD molecular classification.  Inclusion criteria included being conversational in English and identifying as someone who provided care to the person with sCJD. Exclusion criteria included the patient lacking a caregiver candidate or candidate cognitive impairment.  We recruited caregivers from lists of main contacts for patients in the UCSF MAC RPD database who had died from sCJD at least 3 months but no more than 3 years previously. In cases where the primary caregiver was unavailable (e.g., due to death or cognitive impairment) we contacted the next available caregiver listed for the patient. |
| 11. | Method of approach | The interviewer called or (in cases with no number or no working phone number) emailed candidates to share information about the study’s purpose, design and procedures. The interviewer contacted the candidates up to three times in order to make contact. |
| 12. | Sample size | 12 bereaved caregivers for interviews |
| 13. | Non-participation | 23 individuals were contacted before recruitment closed due to the COVID-19 pandemic   - 3 declined, stating that they did not feel they had anything to add, or that they wished not to think about that period of their lives. - 8 we emailed or left messages with but were unable to make contact with |
| Setting |  |  |
| 14. | Setting of data collection | Phone-based. |
| 15. | Presence of non-participants | Non-participants were involved in 0 of the interviews. |
| 16. | Description of sample | Interviews were conducted September 2019 to March 2020 |
| Data collection | | |
| 17. | Interview guide | Our multidisciplinary team (including social scientists, experts in palliative care, a geriatrician, and a neurologist) created and piloted a semi-structured interview guide. Interview domains focused on key experiences along the patient’s disease trajectory; caregiver activities and quality of life; challenges and sources of distress; and things that did or could have helped them to care for the person with sJCD.  This guide had been developed and pilot-tested in a related project regarding palliative care needs in longer-course dementia syndromes. |
| 18. | Repeat interviews | There were no repeat interviews. |
| 19. | Audio/visual recording | With respondent permission we digitally recorded all interviews and had them professionally transcribed. |
| 20. | Field notes | Field notes were taken during the interviews and included among study data. Interviewers also took notes following the conclusion of interviews as early summaries and analyses of the data. |
| 21. | Duration | Interviews lasted a median of 88 minutes (range 41 minutes to 2h 41 minutes) or a mean of 92 minutes (standard deviation 31.5) each. |
| 22. | Data saturation | The research team discussed data saturation at multiple points throughout the project.  The overarching information for which we sought to reach saturation was understanding domains of needs among patients and caregivers. We feel we have reached saturation on major domains.  We did not seek to reach data saturation with use of palliative care (not sampled for); or lived experience (not possible); or domains by type of CJD (not possible with number per type). |
| 23. | Transcripts returned | We did not return transcripts to participants. |
| **Domain 3: analysis and findings** | | |
| Data analysis | | |
| 24. | Number of data coders | 3 – KLH, SBG, CSR |
| 25. | Description of the coding tree | Primary deductive codes were challenges, empirical helps, hypothetical helps, and prion-specific issues. We also coded for diagnostic journey, diagnostic disclosure, caregiving, and end of life/after.  Secondary inductive codes within “prion specific issues” included rarity, rapidity, transmissibility, gravity, autopsy/donation, CJD organizations, and care mismatch.  Secondary inductive codes within challenges and helps included clinical care, caregiving, end-of-life, post-death.  Tertiary codes match the subthemes presented in the tables. |
| 26. | Derivation of themes | An interdisciplinary, international team iteratively reviewed chart data, case summaries, and transcript excerpts throughout data collection to refine analytic approaches and identify preliminary themes.  We conducted both deductive and inductive coding and analysis.  Overall themes were deductively identified based on domains of the interview guide (challenges, empirical helps, hypothetical helps). Iterative discussions and methods of organizing the paper outline and tables led us to determine that the deductive code of “prion specific issues” was also an overarching theme.  Categories and subthemes (e.g. Challenges related to clinical care, specifically, obtaining diagnoses) were inductively identified based on iterative coding and discussion. |
| 27. | Software | ATLAS.ti version 8 |
| 28. | Participant checking | No |
| Reporting |  |  |
| 29. | Quotations presented | Participant quotations are presented to illustrate themes / findings in the manuscript text and associated tables; each quotation is identified with a participant number |
| 30. | Data and findings consistent | In our report we endeavor to maintain consistency between the data presented and the findings |
| 31. | Clarity of major themes | We aimed to clearly present major themes in the findings |
| 32. | Clarity of minor themes | We describe diverse cases and discuss minor themes in the text and table |

**Appendix 2: Interview framework**

*I want to remind you that you can stop the interview at any time. You can also refuse to answer any questions. Again, we will audio-record this interview in order to transcribe it for analysis. Are you willing to proceed with the interview and recording?*

1. **Information about the Caregiver & Patient**

*Objective: Explore the patient’s disease trajectory; CG’s relationship to and the support they provided to patient; explore other people who help the patient and/or CG*.

- First, please tell me how you were related to [____name of patient]?
- Could you tell me when you first started noticing something changing with [patient]?
- Please give me a **brief overview** of what happened between then and when [patient] died.
  - Probe: What other providers or clinics during this period?
  - Who was the captain or quarterback of the patient’s care during this period?

Now I’d like to ask you about your experience as a caregiver.

- What kinds of things did you do with or for [patient] in your day-to-day lives? *[if not clear from overview]*
- Did anyone else help you or your loved one?
  - Who helped?
  - How did they help?
- How would you describe your quality of life at that time? (Social, emotional, physical)
- Did you have any prior experience as caregiver for someone at EOL or with dementia?

1. **Knowledge, disease course & experiences**

*Objective: Explore the degree to which they knew about the disease, the prognosis; how the disease would unfold.*

Please think back to your experience receiving care.

- How did you learn the diagnosis?
- What did provider[s] communicate to you about the disease?
  - Did you receive guidance about what to expect over time?
  - Where did you get this information from? Do you wish it had happened differently?
- What aspects of your and the patient’s lives in this period were difficult or distressing?

*Probe:* Thank you for those answers. I’m going to share you a list of aspects of life with which caregivers sometimes have difficulty. *[[Discussion topic handout]]*

- *Physical: The body, abilities, symptoms*
- *Psychological: The mind, emotions, feelings*
- *Social: Relationships, events, community*
- *Cultural: Community or family traditions, values, preferences*
- *Ethical: Fairness, morals, dilemmas about what is the “right” thing to do*
- *Memory care services: Timing, location, services offered*
- *Behavioral: How they engage with other people or their environment*
- *Spiritual/religious: Meaning, purpose, relationship with God or higher power*
- *Legal: Power of attorney, will, “getting affairs in order”*
- *Financial: Power of attorney, will, “getting affairs in order”*
- *Preparing for end-of-life: Location; types of services (e.g., hospice); loss or grief*
- Did any of these feel difficult or distressing to you? *[Map re timing to EOL.]*
- How did you handle these needs?
  - What helped you?
  - Where did [patient] receive services/support?
  - Who else was involved in these activities? (e.g. family, clinicians)
  - What could have made these difficulties easier or better for you/[patient]?

1. **End of Life Experience**

*Objective: Understand CG’s experience of patient’s end of life period; their needs; whether the needs were met; how/whether they prepared for EOL.*

- Did [patient] have any end-of-life needs that we haven’t discussed? What were they?
- What needs did you have during this period?
  - [Probes: see list above]
- Are there types of services or supports that you wish were available to you or your family during this time?
- Did you and [patient] have an opportunity to discuss their goals for their care? Advanced care planning? Values or preferences for care at the end of life?
  - When did these start?
  - What led you to start these conversations when you did?
    - Do you feel that timing was too early, too late, or just right?
  - Were any clinicians involved in these discussions or preparations? How so? Who?
    - How did you feel about their being involved in this? OR
    - Would you have wanted them to be involved in this? How?
- Were there other things we haven’t discussed that you had to or wanted to take care of before [____] passed? Please describe.
  - Were any clinicians involved in these activities/preparations? How so?
    - How did you feel about their being involved in this? OR
    - Would you have wanted them to be involved in this? How?
- Thinking back, do you wish any of these preparations or activities had gone differently? How? What would have made it easier or better?

1. **Engagement with Palliative Care**

*Objective: Explore to what extent CG/patient engaged w/PC, how, why, and how that shaped the end of life period.*

- Did you engage with anyone from services such as palliative care, symptom mgmt or hospice during this time?
  - *Offer to define palliative care.*
  - If yes: Please tell me about it.
    - Probes: What services provided? Why did you choose to engage them?
    - Would you make the same decision to engage /not engage again? Why/why not?
  - If no: Did you consider engaging with palliative care or related services at any point? Why/why not?
    - Would you make the same decision to not engage PC/related services again? Why/why not?

1. **Caregiver as Expert**

In this final section, we want to get your opinions as an expert. We’re trying to build something to reduce suffering for people like you and [patient].

- What information do you wish you had known when [patient] was first diagnosed? OR
- What advice or information would you give another caregiver starting out on this journey?

*Do you have any questions for me? Is there anything else I should have asked?*

Please complete the following questions for study classification purposes.

1. What **year** were you born? _____________
2. Please mark one: □ Male □ Female □ Other
3. What is your current marital status?

□ Married/partnered

□ Not married, but living together

□ Widowed

□ Divorced or separated

□ Never married

1. How many people live in your household, including yourself? ________

*# of people*

1. Are you Latino or of Hispanic origin or descent?

□ Yes □ No

1. What is your racial background?

Please check **all** that apply:

□ White

□ Black/African-American

□ Asian

□ Native Hawaiian or other Pacific Islander

□ American Indian or Alaska Native

□ Other (please specify): ________________________

1. What is the highest level of school that you have completed, or the highest degree that you have received?

□ Less than high school

□ High school graduate

□ Some college/no degree

□ Associate degree/trade/vocational school

□ College graduate (Bachelor’s degree)

□ Masters/PhD/professional degree

1. Are you a veteran?

□ No

□ Yes

1. Do you have health insurance?

□ No

□ Yes

□ I don’t know

1. If yes, what type of health insurance do you have?

□ Medicare

□ Medicaid/MediCal

□ Private

□ Other

1. How hard is it for you to pay for the very basics like food, housing, medical care, and heating? Would you say it is:

□ Not hard at all □ Somewhat hard □ Very hard

1. Which of these categories is closest to your total annual household income, including income from all family members living with you?

(This includes all income from jobs, pensions, interest investments, Social Security, or other payments.)

□ Less than $20,000

□ $20,000 – < $40,000

□ $40,000 – <$60,000

□ $60,000 – <$80,000

□ $80,000 – <$100,000

□ $100,000 or more
